# Supplementary material for: Glycemic Control and Metabolic Adaptation in Response to High-Fat versus High-Carbohydrate Diets—Data from a Randomized Cross-Over Study in Healthy Subjects
Source: Nutrients. 2021 Sep 23;13(10):3322. doi: 10.3390/nu13103322 (PMC8538379; doi:10.3390/nu13103322)
Supplement: Supplementary file 1 [file nutrients-13-03322-s001.zip › nutrients-1366153-supplementary.pdf]

Supplementary Figure S1. CONSORT diagram for inclusion of study participants.

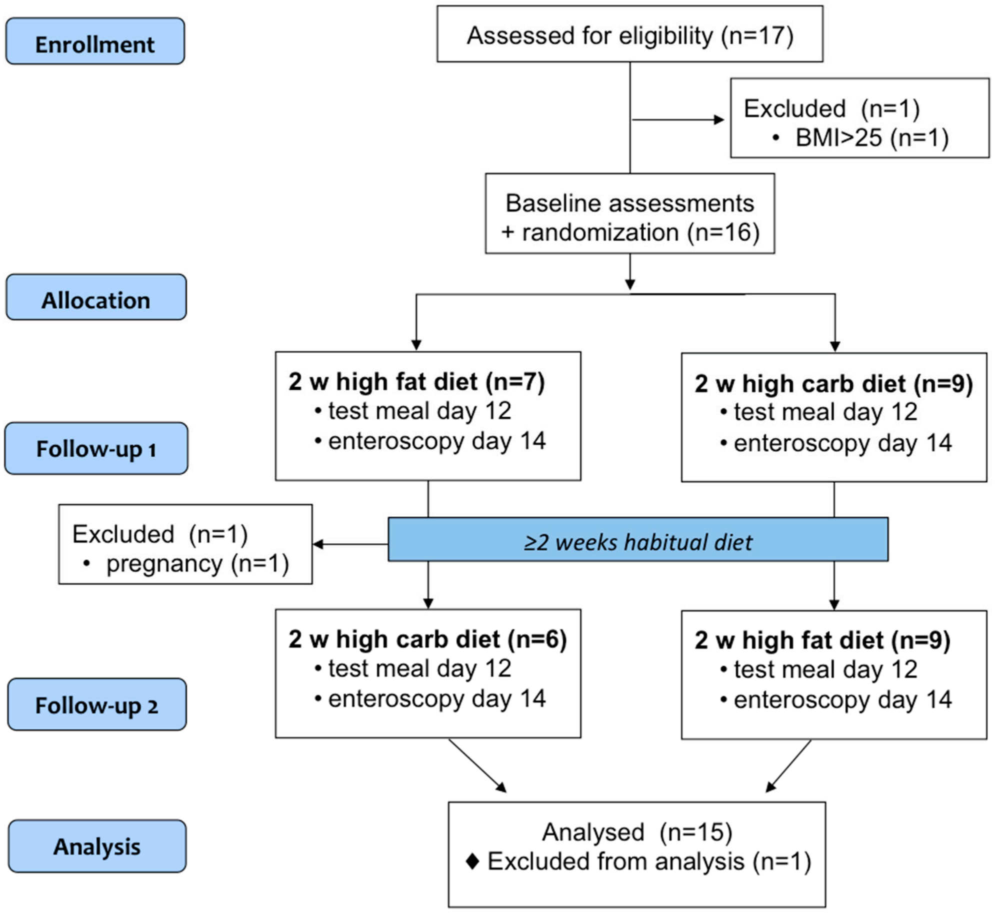

Supplementary Figure S2. Self reported energy intake.

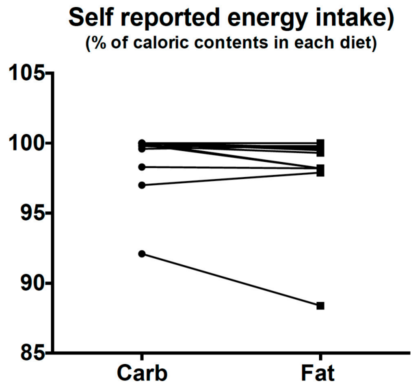

Supplementary Figure S3. Levels of additional metabolites during MMT after HFD and HCD.

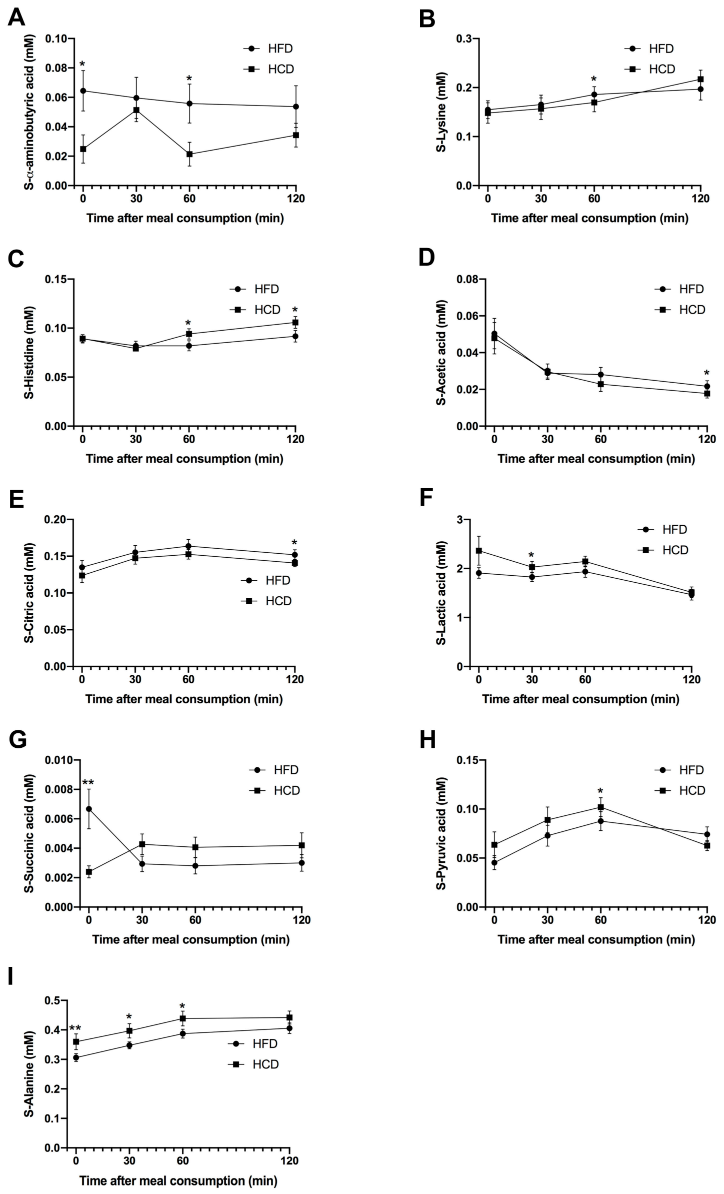

Supplementary Table S1: The dietary composition of the HFD and the HCD.

	2000 kcal/day		2500 kcal/day		3000 kcal/day	
	**HCD**	**HFD**	**HCD**	**HFD**	**HCD**	**HFD**
Energy (kcal)	2034	2025	2514	2520	2996	3014
Protein, g (E%)	100 (20)	97 (19)	127 (21)	118 (19)	152 (21)	139 (19)
Fat, g (E%)	46 (20)	137 (60)	57 (20)	172 (60)	67 (20)	207 (60)
Carbohydrate, g (E%)	299 (60)	107 (21)	365 (59)	131 (21)	437 (59)	207 (21)
Total fiber, g (E%)	13(1,28)	5,6(0,006)	17,1(1,36)	8,1(0,006)	18,7(1,25)	9,4(0,006)
Sucrose, g (E%)	21,9(4,3)	7,3(1,4)	31,5(5,0)	10,2(1,6)	33,8(4,5)	14,9(2,0)
Saturated fat, g (E%)	5,8 (2,5)	37 (16)	9,2 (3,2)	47,4 (16,6)	9,7 (2,8)	57,6 (16,9)
Monounsaterated fat, g (E%)	6,5 (2,8)	22,5 (9,7)	8,6 (3)	32,9 (11,5)	9,8 (2,9)	40,6 (11,9)
Polyunsaturated fat, g (E%)	3 (1,3)	7,1 (3,1)	3,4 (1,2)	11,2 (3,9)	4 (1,2)	14,2 (4,1)
Sodium (mg)*	4318	3667	5129	4331	5928	4732

*Sodium include sodium from industrial cooked meals and most other foods in the menu but excluding salt added to cooked meals in the laboratory kitchen

Supplementary Table S2: Examples of daily menus of the HCD and HFD.

HIGH CARBOHYDRATE DIET DAY	2000 kcal	2500 kcal	3000 kcal
**Breakfast**			
Wholemeal bread (Frökusar, Fazer, Sweden)	80	80	80
Smoked ham	30	60	60
Tomato	70	70	70
Milk 1,5% fat 3 dl (Arla, Sweden)		300	300
**Snack**			
Banana	210	210	210
Drinking yoghurt (Arla, Sweden)			350
**Lunch**			
Chicken thai (Dafgårds, Sweden)	480	480	480
**Snack**			
Apple	125	125	250
Drinking yoghurt (Arla, Sweden)		350	350
**Dinner**			
Goulash soup:			
beef loin	100	100	100
onion	30	30	30
red pepper	30	30	30
garlic	2	2	2
tomato puree	5	5	5
meat broth	250	250	250
potato	150	150	150
Wholemeal bread (Frökusar, Fazer, Sweden)		40	80
Orange juice (Tropicana, Sweden)	330	330	330
**Evening meal**			
Wholemeal bread (Frökusar, Fazer, Sweden)	80	80	80
Smoked ham	30	60	60
Tomato	70		
Blueberry soup (Ekströms, Sweden)	300	300	500

Energy (kcal)	1969	2530	2966
E% protein	18	20	20
E% fat	20	19	18
E% carbohydrates	60	61	62

**HIGH FAT DIET DAY**			
**Breakfast**			
Fried egg	60	60	120
Fried bacon	50	50	90
**Lunch**			
Veal steak (Dafgård, Sweden)	480	480	480
**Dinner**			
Jerusalem artichoke soup:			
jerusalem artichokes	40	40	40
potato	20	20	20
onion	20	20	20
chicken broth	100	100	100
cream 40% fat (Arla, Sweden)	60	60	60
Parmesan cheese (cooked as chips)	20	20	20
Fried pork fillet	100	100	125
Boiled rice (Uncle Ben's,)	87,5	87,5	175
Sauce with a taste of tarragon and mustard:	50	75	75
cream 40% fat (Arla Ltd)	47	70	70
french mustard	3	5	5
Boiled sugar peas	100	100	100
Fruit yogurt 5 % fat (Arla Ltd)		125	
**Evening meal**			
Peanuts		60	100
Foam candy	20	20	20
Energy (kcal)	2011	2516	2996
E% protein	20	19	21
E% fat	59	60	60
E% carbohydrates	21	21	19

Supplementary Table S3: Composition of mixed mini-meal test (MMT).

Energy (kcal)	603
Protein, g (E%)	22,7 (15)
Fat, g (E%)	36,9 (54)
Carbohydrate, g (E%)	45,8 (31)
Total fiber, g (E%)	1,1 (0,004)
Sucrose, g (E%)	5,6 (4,7)
Saturated fat, g (E%)	6,4 (9,4)
Monounsaterated fat, g (E%)	6,5 (9,6)
Polyunsaturated fat, g (E%)	2,1 (3)
Sodium (mg)*	412

*Sodium include sodium from industrial cooked meals and most other foods in the menu but excluding salt added to cooked meals in the laboratory kitchen
